# Quadriceps force generation in patients with osteoarthritis of the knee and asymptomatic participants during patellar tendon reflex reactions: an exploratory cross-sectional study

**DOI:** 10.1186/1471-2474-6-46

**Published:** 2005-09-01

**Authors:** John Dixon, Tracey E Howe

**Affiliations:** 1 Teesside Centre for Rehabilitation Sciences, University of Teesside, The James Cook University Hospital, Middlesbrough, UK; 2 HealthQWest, Glasgow Caledonian University, Cowcaddens Road, Glasgow, UK

## Abstract

**Background:**

It has been postulated that muscle contraction is slower in patients with osteoarthritis of the knee than asymptomatic individuals, a factor that could theoretically impair joint protection mechanisms. This study investigated whether patients with osteoarthritis of the knee took longer than asymptomatic participants to generate force during reflex quadriceps muscle contraction. This was an exploratory study to inform sample size for future studies.

**Methods:**

An exploratory observational cross sectional study was carried out. Two subject groups were tested, asymptomatic participants (n = 17), mean (SD) 56.7 (8.6) years, and patients with osteoarthritis of the knee, diagnosed by an orthopaedic surgeon, (n = 16), age 65.9 (7.8) years. Patellar tendon reflex responses were elicited from participants and measured with a load cell. Force latency, contraction time, and force of the reflex response were determined from digitally stored data. The Mann-Whitney U test was used for the between group comparisons in these variables. Bland and Altman within-subject standard deviation values were calculated to evaluate the measurement error or precision of force latency and contraction time.

**Results:**

No significant differences were found between the groups for force latency (p = 0.47), contraction time (p = 0.91), or force (p = 0.72). The two standard deviation measurement error values for force latency were 27.9 ms for asymptomatic participants and 16.4 ms for OA knee patients. For contraction time, these values were 29.3 ms for asymptomatic participants and 28.1 ms for OA knee patients. Post hoc calculations revealed that the study was adequately powered (80%) to detect a difference between the groups of 30 ms in force latency. However it was inadequately powered (59%) to detect this same difference in contraction time, and 28 participants would be required in each group to reach 80% power.

**Conclusion:**

Patients with osteoarthritis of the knee do not appear to have compromised temporal parameters or magnitude of force generation during patellar tendon reflex reactions when compared to a group of asymptomatic participants. However, these results suggest that larger studies are carried out to investigate this area further.

## Background

Osteoarthritis (OA) of the knee is associated with quadriceps muscle weakness [1,2], muscle dysfunction [3], and proprioceptive impairments [4] that may contribute to the pathogenesis or progression of OA knee by the production of increased joint damage. Minor neuromuscular incoordination has been termed "microklutziness" [5], and may result in impulsive joint loading and an increased heel strike force [1,5,6]. As the quadriceps muscle group is a main stabiliser of the knee joint, muscle weakness or atrophy will of course reduce the amount of protective force generated at the knee joint [1]. In addition, however, if the speed of muscle contraction is also affected and slower, then it will also take longer for protective and stabilising muscle contraction to occur [1,7-9]. Marks et al. [8] observed that the ability to generate force quickly during voluntary muscle contraction was impaired in the quadriceps of OA knee patients. However, due to the protective reflex mechanisms that operate around the knee joint [3,7,10], muscle force generation during reflex reactions may be at least as or more important than voluntary contractions [7,11]. There is an absence of research on quadriceps reflex force generation in OA knee, which may be vital in these protective reflexes. This knowledge may be useful in understanding the aetiology of OA knee. Furthermore, because exercise may improve the speed of force generation [12], and thus may improve knee joint protection [3,9], information on reflex force generation may allow rehabilitative and preventative measures to be improved for this population.

The aim of this study was to investigate whether reflex force generation was impaired in the quadriceps of OA knee patients compared to asymptomatic participants. This was achieved by measuring the standard temporal parameters termed force latency (FL) and contraction time (CT) [13,14], and force during the patellar tendon reflex. FL is the time from tendon tap to onset of quadriceps force generation, and CT is the time from force onset to peak force. Our experimental hypothesis was that there would be a difference in FL, CT and force between the groups. As no published data were available on the parameters of interest in OA knee patients, data from this preliminary study will inform sample size calculations for any future studies.

## Methods

An exploratory observational cross sectional study was carried out in conjunction with an EMG investigation [15].

### Subjects

Ethical approval was granted by the local research ethics committee. Our sample were opportunistic. All subjects gave written and verbal informed consent before taking part in the study. Two groups were tested, symptomatic OA knee patients and asymptomatic subjects. The descriptive characteristics of the subjects are shown in Table 1. OA patients were recruited from South Tees Hospitals NHS Trust, UK, outpatients orthopaedic clinics. Diagnosis of OA knee was made by an orthopaedic surgeon according to the American College of Rheumatology criteria, [16] using clinical signs and symptoms and the presence of osteophytes determined by weight-bearing radiographs. Asymptomatic subjects comprised a convenience sample of volunteers recruited from hospital and university sites and local clubs, and were individuals who reported having no history of knee pain.

**Table 1:** Descriptive characteristics of participants

Group	Age (years) Mean (SD)	Height (m) Mean (SD)	Mass (kg) Mean (SD)	BMI (kg/m^2^) Mean (SD)	Sex (M:F)
Asymptomatic	56.7 (8.6)	1.68 (0.09)	74.4 (10.9)	26.3 (2.7)	8:9
OA	65.9 (7.8)	1.68 (0.10)	82.1 (11.2)	29.0 (2.4)	11:5

Subjects were excluded if they presented with significant cognitive, cardiorespiratory, neurological, or musculoskeletal impairments (excepting OA knee in the patient group) that limited functional ability, or reported use of medication affecting neuromuscular function. One OA knee patient was excluded from the study, as a detectable reflex response could not be elicited, reducing the number of patients from 17 to 16 in that group.

### Materials and procedure

For testing, each subject was seated on an adjustable padded 'quadriceps chair' [17-19] with the posterior aspect of the knee positioned at the front edge of the seat and arms folded across the chest. In patients with unilateral OA, the symptomatic limb was tested. In bilateral OA knee patients and asymptomatic subjects, data were recorded from the dominant limb, which was defined as the limb with which they would kick a ball.

Measurements were made in a non-weight bearing open kinetic chain manner [20,21]. A comfortable non-extensible strap was positioned around the lower leg with the bottom edge at the level of the lateral malleolus. This strap was attached to a light chain that passed horizontally to a calibrated load cell type TC601 (Amber Instruments Ltd, UK) attached to the chair, but adjustable for height and direction. For each subject, the knee joint was carefully set at 90° of flexion, by adjustment of the non-extensible strap, with the chain passing horizontally without slack to the load cell. The load cell output was amplified using a load cell amplifier type LAU 64.200 (Sensor techniques Ltd, UK). The amplified output from this was input to an MP100 workstation (BIOPAC Inc., USA), recorded and stored digitally.

All subjects were blindfolded to minimize external influences. A standard tape of classical music was played to them through headphones. The patellar tendon reflex was elicited by a patellar hammer connected to a pivot on a specially constructed height-adjustable rig [17]. This ensured reproducibility of the force and strike location of each stimulus. The force produced by the hammer was repeatedly tested using the load cell, and was a constant 4.2 N [17]. It was felt that this force was sufficient to elicit a reflex response, and that higher force could cause pain to patients. A vibration sensor (Maplin Electronics, UK) attached to the hammer arm and connected to the MP100 workstation produced a square wave time marker on the recording on impact. Ten taps to the patellar tendon were delivered at random time intervals (10 to 20 s apart) to reduce habituation or fatigue [22,23]. Data were sampled at 2048 Hz using a physiological data acquisition system (BIOPAC Inc., USA) comprising the MP100 workstation with a high level transducer HLT100 and dedicated analysis software (AcqKnowledge 3.5.3).

Force latency, defined as the time from tendon tap to onset of force generation, and contraction time, defined as the time from force onset to peak force, were determined from each response using the digital outputs of the AcqKnowledge software. The peak force of each reflex response was also recorded. For each subject the resting tension level was taken into account by subtraction when calculating the force output. The mean values of the ten responses per subject for each variable were analysed using SPSS version 9.0. As statistical analysis showed that the data for FL and CT and force did not follow a normal distribution, (Shapiro-Wilk test p < 0.05), the between group comparisons were made with the non-parametric Mann-Whitney U test and non-parametric approximate 95% confidence intervals were calculated. In addition, to assess the measurement error and precision of the FL and CT measurements, the within-subject standard deviations were calculated using the Bland and Altman method [24]. Using this, the difference between an observed value and a participant's true value is expected to be less than two standard deviations for 95% of observations. Post hoc power calculations were carried out to evaluate whether the study was sufficiently powered, and to inform future research.

## Results

The main results are shown in Table 2. No significant difference was found between the groups for FL or CT (Mann Whitney U test, FL p = 0.47, CT p = 0.91). The Hodges-Lehmann median difference between the groups was -5 ms (95% CI -32 to 8 ms) for FL, and 1 ms (95% CI -16 to 17.5 ms) for CT.

**Table 2:** Median (approximate 95% confidence interval) values for force generation parameters

Group	FL (ms)	CT (ms)	Force (N)	Force/Body Mass (N/kg)
Asymptomatic	62.6 (54.9–87.2)	149.8 (141.5–171.3)	5.3 (2.2–16.5)	0.08 (0.03–0.20)
OA	64.6 (54.0–109.9)	146.4 (137.1–167.3)	4.9 (1.1–18.5)	0.06 (0.01–0.25)

FL, force latency; CT, contraction time

Using the Bland and Altman method to calculate the within-subject standard deviations for FL and CT, no relationships were seen between means and standard deviations and hence this method was valid. For 95% of measurements the difference between a single observed value and a participant's true value for FL is said to be at most two standard deviations [24], i.e. 27.9 ms for asymptomatic participants and 16.4 ms for OA knee patients. For CT, these values were 29.3 ms for asymptomatic participants and 28.1 ms for OA knee patients.

To evaluate whether the study was adequately powered to detect differences between the groups in FL and CT, post hoc calculations were carried out with a significance level of 0.05. Due to the measurement error reported above, it was decided to use a value of 30 ms as the difference to be detected, as any smaller value could be less than the expected measurement error or precision of the readings. The calculations showed that 17 participants in each group were sufficient to detect a difference of 30 ms in FL at 80% power. Hence this study was sufficiently powered to detect a difference of that magnitude for this variable. However, 28 participants in each group were required to provide 80% power for CT, and this study with 17 participants had only 59% power.

The Mann-Whitney U test did not reveal any statistically significant difference between the groups in the absolute peak force of the reflex response (p = 0.72) or peak force of the reflex response when normalised to body mass (p = 0.54).

## Discussion

We measured parameters of reflex force generation of the quadriceps in OA knee patients and asymptomatic participants during the patellar tendon reflex response. There were no statistically significant differences between the groups for force latency, contraction time, or force. This is an important finding as it has been reported that quadriceps muscle contraction is slower in OA knee patients than in asymptomatic individuals [9], despite the fact that this only appears to have been studied once [8], and not during reflex responses. The study by Marks et al. [8] reported that the ability to generate voluntary force quickly was impaired in the quadriceps of OA knee patients compared to young participants, but a comparison with participants of a similar age was not made. As there is evidence that a slowing of muscle contraction occurs with age [10], the difference in age between the groups in that study may have been a confounding variable. In the study here, both the OA knee and asymptomatic group comprised participants ranging from middle to old age. The fact that no significant difference was found between the groups, paired with the known significant correlation of these parameters with age [10] indicates that age-related physical changes may have more of an effect than knee joint pathology on these force generation parameters. In support of the validity of our results, our data are similar to published values, for example Burke et al. [13] reported a mean FL of 77 ms for a group of old participants, mean age 74 years.

These parameters have relevance to rehabilitation and patient care because any impairment, even only if age-related, in the time taken to initiate and then generate muscle force will increase the time taken to produce movement or stability and could impair protective reflexes [3,10]. Marks et al. [9] discuss how an improvement in the rate of force generation may reduce the rate and amount of damage to the knee joint. Strength training can increase the rate of voluntary force development [25,26], and reduce the time to peak force [27]. One of the mechanisms by which this occurs is the increase in tendon stiffness that occurs with strength training [12]. Therefore, importantly for patient care, strengthening exercises may improve contractile mechanisms that become impaired with age. These improvements may be one of the reasons why exercise is beneficial in OA knee [28] and why moderate exercise is associated with a reduced risk for OA in later life [29]. Longitudinal studies evaluating these parameters, and their associations with functional ability and pain and muscle strength would be beneficial.

However, the results of this study should be viewed with caution. The lack of a significant difference between the groups could be attributable to several explanations. These could include the small sample size, the heterogenous characteristics of the participants, other underlying conditions in the participants, and the stimulus and instrumentation used. Firstly, the results could possibly be due to a lack of study power. From our results, this study had adequate power (80%) to detect a difference of 30 ms between the groups in FL, but had only 59% power to detect this in CT. Further research with a larger sample size is therefore recommended, and this study provides data that will inform sample size calculations for future studies. To avoid making a type II error from the results observed, future studies should use a much larger total sample size, possibly necessitating a multicentre approach.

It is possible that the OA knee patients in this study could be a heterogenous group, as the general OA knee population is understood to display considerable heterogeneity [1]. No attempt was made to categorize patients in terms of aspects of OA knee classification, such as duration or level of symptoms. If this was the case in future studies, sub-group analysis could reveal whether specific sub-groups of the OA knee population do exhibit an impairment in FL and CT that could be clinically important. In addition, an assumption was made that the asymptomatic participants did not have radiographic OA. Although radiographs were not taken from the asymptomatic participants for ethical reasons, it is possible that they could have exhibited asymptomatic radiographic changes at the knee joint [30], and that this may possibly have affected the physiological responses. Future studies could also take into consideration gender and anthropometric variables, such as height and the length of the spinal reflex, that could also affect FL.

The effect of ageing on muscle function is known, but much of this effect may be indirect, i.e. it may actually be the effect of the inactivity and atrophy that may occur with ageing, and some people remain active and others do not. We did not attempt to quantify the physical activity or fitness levels of participants, or to match these between the groups. In addition, it is well known that the prevalence of other medical conditions such as neurological disease increases with age, and some participants may have had undiagnosed or sub-clinical conditions of this nature, although it is anticipated that this would have been similar in both groups.

We could possibly have obtained larger and perhaps quicker responses using a greater stimulus, as reflex response amplitude increases in a sigmoidal manner with increasing stimulus force [31,32]. The force generated in the reflex response does have an effect on FL, because as contraction force increases, electromechanical delay reduces [33,34]. In one patient, a reflex response could not be detected, and this was presumed to be due to the marked knee effusion exhibited. It is possible that a greater stimulus force could have elicited a reflex reponse in that patient. However patient comfort was an issue and reflex responses should only be compared when stimulus intensities are the same [35]. However, the repeatability of this equipment was tested and found to be constant (4.2 N) [17].

The ability to magnify or resolve traces has been shown to affect the observed electromechanical delay [36], and will probably affect values observed for all temporal parameters such as FL and CT. In evaluating the Bland and Altman precision or measurement error in FL and CT from the ten responses of each participant, we found that a single measurement could differ from a participant's true value by almost 30 ms. This may be due to the known variability in the force of the patellar tendon reflex response [23]. However there are no similar data available for FL or CT for comparison. Interestingly, this level of measurement error is far larger than the non-significant differences between the groups. Our data evaluation used modern digital methods rather than pen recordings as used in some earlier studies [13], and hence may have greater accuracy.

Finally, the open kinetic chain reflex muscle contraction tested here is a standard functional test used clinically which provides important quadriceps specific information [37]. However, this may not be representative of normal functional movement that tends to occur in the weight bearing closed kinetic chain manner [20,21]. Future studies should attempt to use both open and closed kinetic chain testing.

Much of our understanding of muscle dysfunction in OA knee remains speculative [1]. Recent studies continue to shed new light on this area, showing for example that not all OA knee patients exhibit the expected arthrogenous muscle inhibition [38], and that quadriceps strengthening may not benefit patients with malaligned knees [39]. We believe that this study adds to the evidence base and informs an area for further research that may be important and may have clinical relevance. The results of the only published paper on this topic [8] may have been affected by age differences between the two groups, but also by technological limitations. No further studies appear to have followed up this work, and this should be rectified.

## Conclusion

Our experimental hypothesis in this exploratory study was that there would be a difference in FL, CT, or force between the groups during the patellar tendon reflex response. However, the results showed no statistical difference between the groups in these variables. This appears to be the first study to compare reflex FL and CT production in OA knee patients with that of asymptomatic participants. These findings provide some evidence that the time taken to generate reflex force is not impaired in OA knee patients compared to a group of similarly aged asymptomatic participants, but further research is required in this area.

## Competing interests

The author(s) declare that they have no competing interests.

## Authors' contributions

Both authors participated in the design of the study, the statistical analysis, and the drafting, progress and revision of the manuscript. JD collected the data and carried out the data extraction and analysis.

## Pre-publication history

The pre-publication history for this paper can be accessed here:


